# Effect of Central Venous Nutrition Infusion, Intravenous Fat Emulsion, and Therapeutic Agents on the Particle Size of Fat Emulsion for Simultaneous Administration of Three Drugs

**DOI:** 10.1159/000526329

**Published:** 2022-08-05

**Authors:** Mika Onishi, Yoshihito Koyama, Akemi Hagika, Sachiko Omotani, Yasutoshi Hatsuda, Naoyoshi Maezaki, Michiaki Myotoku

**Affiliations:** ^a^Faculty of Pharmacy, Osaka Ohtani University, Tondabayashi, Japan; ^b^Department of Pharmacy, Higashi-Sumiyoshi Morimoto Hospital, Osaka, Japan

**Keywords:** Central venous nutrition infusion, Fat emulsion, Injection, Therapeutic agent

## Abstract

**Background:**

In Japan, therapeutic agents are often administered through the side tube of a central venous line or mixed with a total parenteral nutrition (TPN) infusion. This is expected to result in the mixture of three drugs in the infusion line: the infusion product for TPN, the fat emulsion, and the therapeutic agent. Therefore, we investigated whether various therapeutic agents affect the particle size of the fat emulsion.

**Methods:**

In model of administration A, the TPN infusion formulation was administered through the main tube, and the fat emulsion and therapeutic agents were simultaneously administered through the side tube; 21 therapeutic agents were used. In model of administration B, the TPN infusion formulation mixed with therapeutic agents was administered through the main tube, and the fat emulsion was simultaneously administered through the side tube; 20 therapeutic agents were used. The number of fine particles for each particle size range in the mixed solution was measured over time using a light-shielding automatic fine-particle measuring device.

**Results:**

In model A, the number of fine particles in the fat emulsion changed rapidly for five therapeutic agents and slowly for two therapeutic agents. In model B, this change occurred drastically for five therapeutic agents and slowly for one therapeutic agent.

**Conclusions:**

Some therapeutic agents may contribute to fat particle aggregation. Therefore, these therapeutic agents should not be concurrently administered with fat emulsions.

## Introduction

Intravenous fat emulsion (fat emulsion) administration during total parenteral nutrition (TPN) is very important as a heat source as well as in preventing essential fatty acid deficiency, liver dysfunction, and fatty liver development [1, 2]. However, since fat emulsion formulations consist of soybean oil emulsified by egg yolk lecithin, the effects of divalent cations, amino acids, and pH can cause emulsion instability and particle coarsening [3, 4, 5, 6, 7]. Additionally, it poses a risk of embolization because of coarse fat emulsion particles [8, 9]. Therefore, TPN infusion products containing carbohydrates, electrolytes, amino acids, and fat emulsions cannot be administered together as a single dose because the coarsening of fat emulsion particles could occur [10]. Although administering it alone through the peripheral intravenous route is recommended, fat emulsion can be administered through a lateral tube in the central venous line during TPN, in cases where securing a peripheral intravenous route is difficult [10, 11]. However, these only apply to infusion formulations for TPN mixed with high-calorie total vitamins and trace element formulations and do not apply to other therapeutic agents.

The 3rd edition of the Guidelines for Intravenous and Enteral Nutrition [2] states that the rate of fat emulsion administration should be ≤0.1 g kg^−1^ h^−1^ [12, 13]. At this rate, administering 250 mL of 20% fat emulsion to a patient weighing 50 kg will take ≥10 h. Therefore, other therapeutic agents may be simultaneously administered through the same infusion line while fat emulsion is being administered through a lateral tube. In fact, in Japan, therapeutic agents are often administered through the lateral tube of the central venous line or mixed with TPN infusion products. This may lead to the mixture of TPN infusions, fat emulsion, and therapeutic agents in the infusion line. We have reported that the coarsening of fat emulsion particles occurs in an experimental model wherein a certain antimicrobial agent is simultaneously administered with TPN infusions and fat emulsion through an infusion line [14].

Therefore, we assumed that the fat emulsion and the therapeutic agent are administered from the side tube of the central intravenous line while the infusion preparation for TPN is administered, and the fat emulsion is administered from the side tube of the infusion line for TPN infusion preparation mixed with the therapeutic agent. We investigated the effect of simultaneously administering three drugs on fat emulsion particle size. Specifically, with the mixture of three drugs, we measured fat emulsion particle size over time using a light-shielded automatic particle counter, and the pH change was also measured and evaluated.

## Experimental Materials and Methods

### Materials

A 20% intravenous fat emulsion (Intralipos® infusion solution 20% 100 mL; Otsuka Pharmaceutical Factory, Inc., Tokushima, Japan) was used as the fat emulsion, and glucose, electrolytes, amino acids, multivitamins, and trace element solutions were used as the high-calorie TPN infusion preparation (Elneopa® NF2 infusion 1,000 mL; Otsuka Pharmaceutical Factory, Inc.). Twenty-one therapeutic agents (Table 1) were administered from the side tube of the central venous line. Therapeutic agents administered through the lateral tube of the central venous line were dissolved in 100 mL of 5% glucose solution (Otsuka glucose solution 5% 100 mL; Otsuka Pharmaceutical Factory, Inc.). Twenty therapeutic agents (Table 1) were mixed with the TPN infusion solution. Lyophilized therapeutic agents were dissolved in an appropriate amount of 5% glucose solution. Therapeutic agents used in the experiment were selected from those that were actually prescribed or that the physician had inquired about from the pharmacist.

### Sample Preparation

**Fig. 1:**
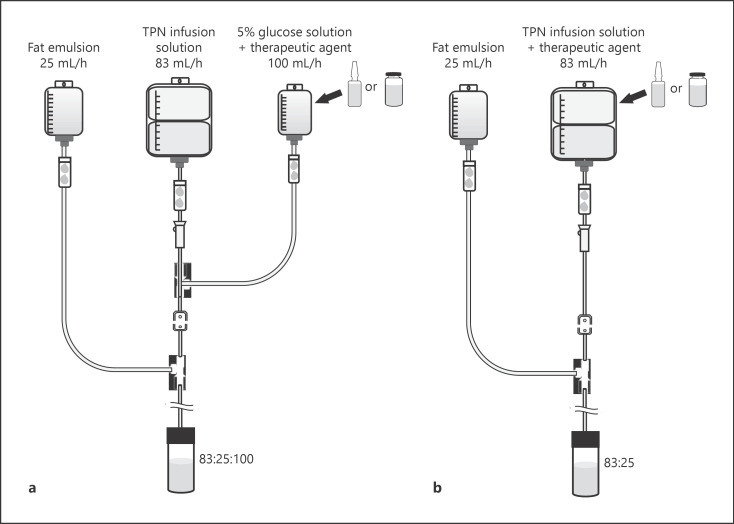
**a, b** Image of the administration model.

Figure 1 shows the administration model used in the study. The daily infusion volume was assumed to be 2 L of TPN infusion solution from the main tube (administration rate: 83 mL/h) and 250 mL of 20% intravenous fat emulsion from the side tube (administration rate: 25 mL/h). The therapeutic agent administered through a lateral tube was presumably administered with 100 mL of 5% glucose solution dissolved in the mixture (administration rate: 100 mL/h). The therapeutic agent mixed with the TPN infusion product was assumed to be administered directly into the TPN infusion product or diluted in a minimal amount of 5% glucose solution and mixed into the TPN infusion product. The therapeutic agent was set to the dosage shown in Table 1, which was assumed to be an actual clinical dosage.

Specifically, the fat emulsion and the therapeutic agent administered through the side tube of the central venous line (model A) had an 83:25:100 ratio, which pertains to the TPN infusion product, the fat emulsion, and the therapeutic agent dissolved in 100 mL of 5% glucose solution, respectively. The fat emulsion administered through the side tube of the TPN infusion product mixed with the therapeutic agent (model B) had an 83:25 ratio, which pertains to the TPN infusion product mixed with the therapeutic agent and the fat emulsion, respectively. To generate the different samples, the mixtures were weighed into 100-mL glass vials (Mighty Vials; Maruemu Co., Ltd., Osaka, Japan) on a clean bench and allowed to stand. In model A, a 5% glucose solution without the therapeutic agent was used as the control (control A). In model B, an unmixed TPN infusion solution was used as the control (control B). The fat emulsion dose was designed to be administered to a patient weighing 50 kg at the guideline-recommended dose rate of 0.1 g kg^−1^ h^−1^ [2].

### Determining the Number of Insoluble Particles in a Sample

The number of insoluble particles was measured in accordance with the 17th edition of the *Japanese Pharmacopoeia*, “Insoluble Particle Test Method for 6.07 Injectable Drugs, Method 1: Light-shielded Particle Counting Method” [15] using a light-shielded automatic measuring device (KL-04A; Rion Co., Ltd., Tokyo, Japan). The measurement of particle sizes was done in the ranges of 2–5, 5–10, 10 μm or larger, and the number was measured. Each of the three sample measurement volumes was 5 mL, and the average value was taken as the measured value for this study.

After preparation, 0.3 mL of each sample was collected. Thereafter, it was diluted with 80 mL of ultrapure water and used for the measurement of the number of particles in the solution. The ultrapure water used for dilution was filtered through a 0.22-μm filter and left for 2 days for degassing. Additionally, a light-shielded automated particulate analyzer was used to confirm that no insoluble particulates existed in the ultrapure water. All instruments used were washed with ultrapure water to remove any foreign matter adhering to the instruments. The number of particles in the solution was measured immediately after preparation and after 1, 3, 6, and 9 h because the fat emulsion particle size increased even in the controls, which were measured in a similar fashion. The samples were stored at 25 ± 1°C in the dark, and the measurements were performed at the same temperature (approximately 25°C). Severe change was defined as a ≥200% increase in the number of particles ranging 2–5 μm, 1 h after preparation compared to that of the controls; slow change was defined as a ≥200% increase in the number of particles ranging 2–5 μm, 3 h after preparation compared to that of the controls.

### Measuring pH Change

The pH of each sample was measured, immediately and in a 24-h timelapse after preparation, using a benchtop pH meter (SevenCompact pH meter S210; Mettler Toledo Co., Ltd., Tokyo, Japan).

## Results

### Change in the Number of Insoluble Particles

**Fig. 2:**
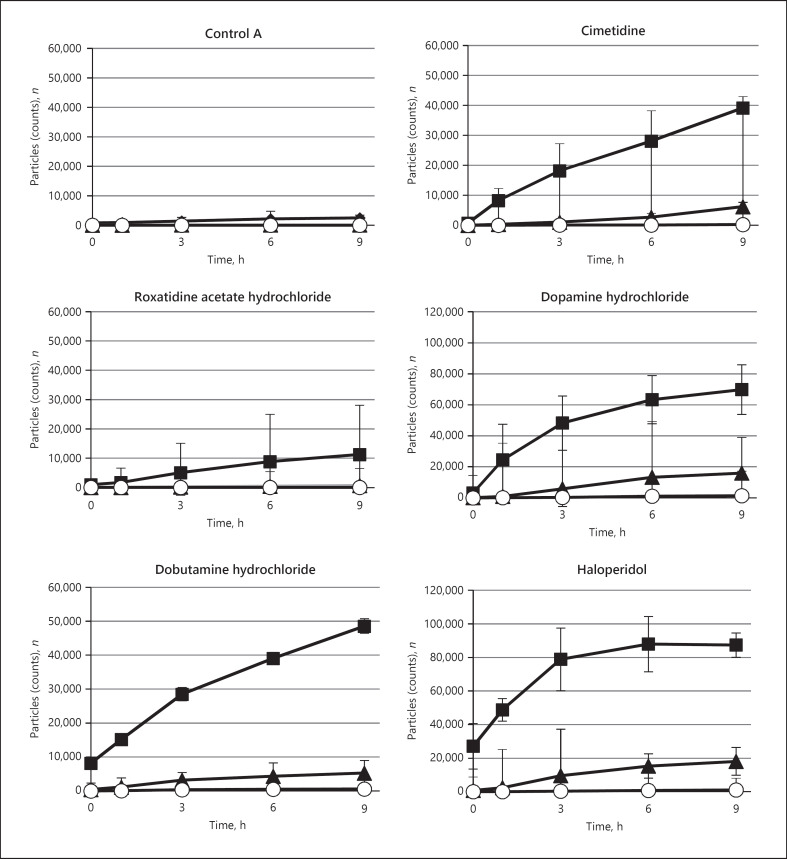
Change with time in the number of insoluble particles for each therapeutic agent that underwent severe changes when the fat emulsion and the therapeutic agent were administered through the side tube of the central venous line. ◼: 2–5 μm, ▲: 5–10 μm, ⚪: 10 μm or more, mean ± SD, *n* = 3.

**Fig. 3:**
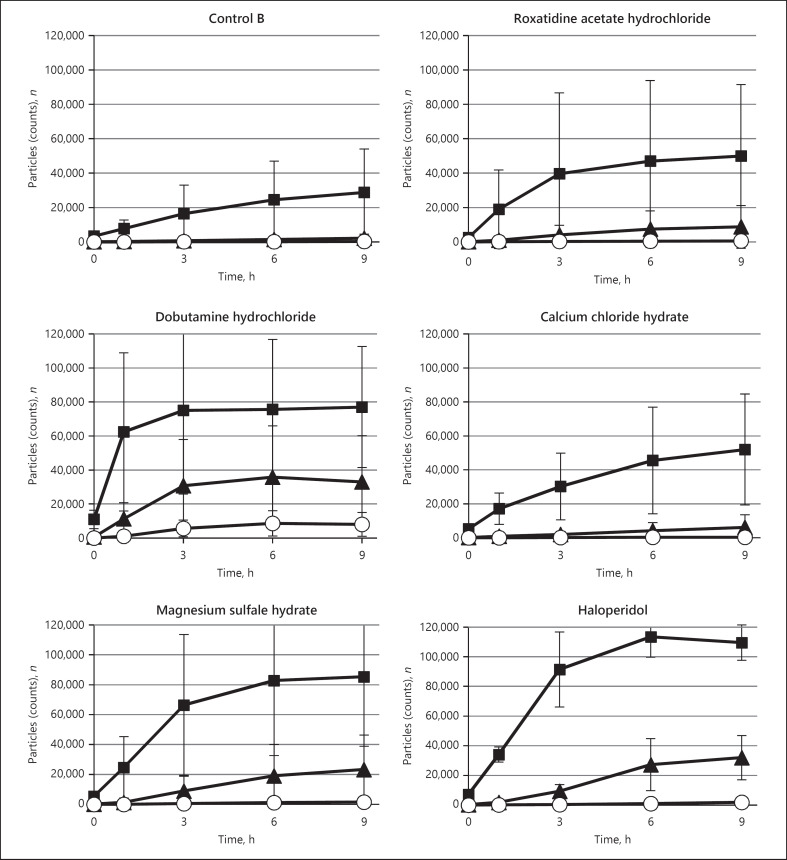
Change with time in the number of insoluble particles for each therapeutic agent that underwent severe changes when the fat emulsion was administered through the side tube of the TPN infusion solution mixed with the therapeutic agent. ◼: 2–5 μm, ▲: 5–10 μm, ⚪: 10 μm or more, mean ± SD, *n* = 3.

The presence or absence of changes in fat emulsion particle size in models A and B is shown in Table 2. In addition, the temporal changes in the number of insoluble particles in the samples that underwent severe changes are shown by particle size in Figures 2 and 3 for models A and B, respectively.

In model A, the five therapeutic agents that caused severe changes were cimetidine, roxatidine acetate hydrochloride, dopamine hydrochloride, dobutamine hydrochloride, and haloperidol. The two therapeutic agents that caused slow changes were carbazochrome sodium sulfonate hydrate and metoclopramide. In model B, the five therapeutic agents that caused severe changes were roxatidine acetate hydrochloride, dobutamine hydrochloride, haloperidol, calcium chloride, and magnesium sulfate. The one therapeutic agent that caused a slow change was carbazochrome sodium sulfonate hydrate.

### Measuring pH Change

For each sample, the pH values immediately and 24 h after preparation in models A and B are shown in Table 3. In model A, the pH of the samples was between 5.30 and 5.53, which was almost the same as that of the control (pH 5.38). At 24 h after preparation, the pH of the samples was between 5.29 and 5.54, which was also almost the same as that of the control (pH 5.37). In addition, there was no significant pH change in the solutions immediately and 24 h after preparation. In model B, the pH of the samples was between 5.29 and 5.36, which was also almost the same as that of the control (pH 5.36). At 24 h after preparation, the pH of the samples was between 5.29 and 5.35, which was not significantly different from that of the control (pH 5.39). There was also no significant pH change in the solutions immediately and 24 h after preparation. The pH values of the solutions were similar for models A and B. Although the data were not presented in this study, some drugs were found to have no visual change in appearance despite being the therapeutic agents that caused severe changes in administration models A and B.

## Discussion

Fat emulsions consist of homogeneously dispersed fat particles in a water layer. Factors causing fat particle aggregation include temperature [16], pH, sugars, electrolytes, amino acids, and plasma expanders [6]. In addition, the presence of electrolytes in the infusion solution, especially that of divalent cations, has a significant effect on the stabilization of fat particles, while high concentrations of monovalent cations have an effect on the coarsening of fat particles [17]. In this study, insoluble particle count and pH change were observed in a formulation change. Therefore, there was a risk of being unable to directly observe appearance change due to the combination of the fat emulsion and other components. The change in the three-in-one formulation, which is a mixture of sugar/electrolyte infusion, amino acid infusion, and fat emulsion, could not be visually confirmed in a previous report [18]. Although the data were not presented in this study, some drugs were found to have no visual change in appearance despite being the therapeutic agents that caused severe changes in models A and B. In the formulation of fatty emulsions with therapeutic agents, judging visual changes in appearance was difficult. The method using a light-shielded automatic particle counter, which measures the number of particles by particle size, may thus be an extremely useful method.

In this study, we examined the combination changes of 21 therapeutic agents in model A and 20 therapeutic agents in model B. Since the purpose of the study was to clarify the formulation changes in the fat emulsion and each therapeutic agent, 5% dextrose solution, which does not affect fat emulsion particle size, was used as the infusion formulation for dissolving the lyophilized product and administering it through the side tube [10]. Dopamine hydrochloride and dobutamine hydrochloride are administered without dilution using syringe pumps in many hospitals. In this study, the dose of the drug per hour was the standard dose used in clinical practice, but the solutions were diluted to ensure that the conditions of administration were identical.

Cimetidine, roxatidine acetate hydrochloride, dopamine hydrochloride, dobutamine hydrochloride, carbazochrome sodium sulfonate hydrate, and metoclopramide, which affected the fat emulsion particle size observed in this study, have relatively basic functional groups according to their chemical structures and are thought to exist in a positively charged state under the pH conditions used in this study. The fat emulsion uses egg yolk lecithin, an amphoteric surfactant, as an emulsifier, and the fat particle surface was weakly and negatively charged. Therefore, mixing it with these therapeutic agents may have caused charge bias in the fat particles, which destabilized and aggregated the fat particles. Haloperidol is highly polar because of the presence of fluorine and chlorine atoms in its chemical structure. This may have destabilized the fatty particles by causing a bias in electric charge, resulting in particle coarsening. In addition, calcium chloride and magnesium sulfate, which have divalent cations, weaken the electrical repulsive force between fat particles, causing fat particles to progress from flocculation to creaming and oil droplet separation, which leads to emulsion destruction [5, 6]. Many of the therapeutic agents that have caused formulation changes have highly polar chemical structures and cations. Furthermore, the combination of the fat emulsion and the TPN infusion formulation, which contains a mixture of sugars, amino acids, and electrolytes, may have contributed to the coarsening of the fat emulsion particles.

Among the drugs tested in both models, five (cimetidine, roxatidine acetate hydrochloride, dopamine hydrochloride, dobutamine hydrochloride, and haloperidol) caused severe changes and two (carbazochrome sodium sulfonate hydrate and metoclopramide) caused slow changes in model A. Meanwhile, five (roxatidine acetate hydrochloride, dobutamine hydrochloride, haloperidol, calcium chloride hydrate, and magnesium sulfate hydrate) caused severe changes and one (carbazochrome sodium sulfonate hydrate) caused a slow change in model B. The difference between the two groups may be because of the differing number of therapeutic agents used during mixture. Model A contained a larger amount of the therapeutic agent per unit dose and was more concentrated.

To confirm pH effect, we measured pH change immediately and 24 h after preparation for each sample. The results showed that the TPN infusion formulation was highly buffered, and the mixture of each therapeutic agent was almost the same as that of the control immediately and 24 h after preparation, with no change observed. Although pH is a factor in fat particle aggregation [6], no pH change was observed in all the drugs. Therefore, pH was not considered to be involved in the formulation change in the present experiment.

The results suggested that in addition to electrolytes and amino acids, some therapeutic agents may contribute to fat particle aggregation. When administering fat emulsion as described by model A, the therapeutic agents that have caused severe changes in the size of fat particles when employing this model should not be administered through the side tube concomitantly with the fat emulsion. If it is necessary to administer the aforementioned therapeutic agent, a separate route should be used for administration. If this is difficult, avoid administering the fat emulsion at the same time and flush the route with saline before and after administration of the therapeutic agent. When infusion products for TPN are administered together with the fat emulsion, do not mix the therapeutic agent that induced severe changes when using model B.

## Statement of Ethics

An ethics statement was not required for this study type, and no human or animal subjects or materials were used.

## Conflict of Interest Statement

The authors have no conflict of interest.

## Funding Sources

The authors have no funding to disclose.

## Author Contributions

Mika Onishi participated in the design of the study and drafting of the manuscript. Yoshihito Koyama and Akemi Hagika analyzed the experimental data. Sachiko Omotani, Yasutoshi Hatsuda, and Naoyoshi Maezaki participated in the interpretation of data. Michiaki Myotoku participated in the conception of the study and revision of the manuscript. All the authors approved the final version of the manuscript.

## Data Availability Statement

All data generated or analyzed during this study are included in this article. Further inquiries can be directed to the corresponding author.

## Figures and Tables

**Table 1:** Therapeutic agents used in the experiment and their mixing amounts

Generic name	Product name	Company	Mixing amount	
			model A	model B
Famotidine	Gaster^®^	LTL Pharma Co., Ltd.	10 mg	10 mg
Cimetidine	Tagamet^®^	Sumitomo Dainippon Pharma Co., Ltd.	200 mg	400 mg
Ranitidine hydrochloride	Zantac^®^	GlaxoSmithKline PLC	50 mg	100 mg
Roxatidine acetate hydrochloride	Altar^®^	ASKA Pharmaceutical Co., Ltd.	75 mg	75 mg
Betamethasone sodium phosphate	Rinderon^®^	Shionogi & Co., Ltd.	2 mg	4 mg
Prednisolone sodium succinate	Predonine^®^	Shionogi & Co., Ltd.	10 mg	20 mg
Hydrocortisone sodium succinate	Solu-Cortef^®^	Pfizer Japan Inc.	100 mg	200 mg
Methylprednisolone sodium succinate	Solu-Medrol^®^	Pfizer Japan Inc.	250 mg	250 mg
Dexamethasone sodium phosphate	Decadron^®^	Aspen Japan Co., Ltd.	6.6 mg	6.6 mg
Dopamine hydrochloride	Inovan^®^	Kyowa Hakko Kirin Co., Ltd.	100 mg	200 mg
Dobutamine hydrochloride	Dobutrex^®^	Kyowa Yakuhin Co., Ltd.	100 mg	200 mg
Furosemide	Lasix^®^	Sanofi Co., Ltd.	20 mg	20 mg
Carbazochrome sodium sulfonate hydrate	Adona^®^	Nipro Corporation	50 mg	50 mg
Tranexamic acid	Transamin^®^	Daiichi Sankyo Co., Ltd.	250 mg	250 mg
Haloperidol	Serenace^®^	Sumitomo Dainippon Pharma Co., Ltd.	5 mg	10 mg
Metoclopramide hydrochloride	Primperan^®^	Nichi-Iko Pharmaceutical Co., Ltd.	10 mg	20 mg
Octreotide acetate	Sandostatin^®^	Novartis Pharma Co., Ltd.	100 µg	200 µg
Lansoprazole	Takepron^®^	Teva Takeda	30 mg	
Granisetron hydrochloride	Kytril^®^	TAIYO Pharma Co., Ltd.	3 mg	
Ramosetron hydrochloride	Nazea^®^	LTL Pharma Co., Ltd.	0.3 mg	
Omeprazole sodium hydrate	Omepral^®^	AstraZeneca Co., Ltd.	20 mg	
Heparin sodium	Heparin sodium	Novartis Pharma Co., Ltd.		10,000 units
Calcium chloride hydrate	Calcium chloride	Otsuka Pharmaceutical Factory, Inc.		400 mg
Magnesium sulfate hydrate	Magnesium sulfate	Otsuka Pharmaceutical Factory, Inc.		20 mEq

**Table 2:** Change in insoluble particle size of fat emulsion

Generic name	Change in particle size	
	model A	model B
Famotidine	−	−
Cimetidine	•	−
Ranitidine hydrochloride	−	−
Roxatidine acetate hydrochloride	•	•
Betamethasone sodium phosphate	−	−
Prednisolone sodium succinate	−	−
Hydrocortisone sodium succinate	−	−
Methylprednisolone sodium succinate	−	−
Dexamethasone sodium phosphate	−	−
Dopamine hydrochloride	•	−
Dobutamine hydrochloride	•	•
Furosemide	−	−
Carbazochrome sodium sulfonate hydrate	○	○
Tranexamic acid	−	−
Haloperidol	•	•
Metoclopramide hydrochloride	○	−
Octreotide acetate	−	−
Lansoprazole	−	
Granisetron hydrochloride	−	
Ramosetron hydrochloride	−	
Omeprazole sodium hydrate	−	
Heparin sodium		−
Calcium chloride hydrate		•
Magnesium sulfate hydrate		•

Model A: the fat emulsion and the therapeutic agent administered through the lateral tube of the central venous line; model B: the fat emulsion administered through the side tube of the TPN infusion product mixed with a therapeutic agent; •, the number of insoluble particles in the range of 2–5 µm increased by 200% or more compared to control at 1 h after preparation; o, the number of insoluble particles in the range of 2–5 µm increased by 200% or more compared to control at 3 h after preparation (other than •); −, no change in the insoluble particle size; gray area: not done in this experiment.

**Table 3:** The pH values immediately after mixing and after 24 h of preparation

Generic name	Model A		Model B	
	0 h	24 h	0 h	24 h
Control	5.38	5.37	5.36	5.39
Famotidine	5.36	5.37	5.30	5.30
Cimetidine	5.37	5.37	5.30	5.30
Ranitidine hydrochloride	5.40	5.42	5.35	5.34
Roxatidine acetate hydrochloride	5.38	5.39	5.34	5.34
Betamethasone sodium phosphate	5.40	5.48	5.31	5.29
Prednisolone sodium succinate	5.39	5.40	5.29	5.33
Hydrocortisone sodium succinate	5.43	5.43	5.29	5.33
Methylprednisolone sodium succinate	5.53	5.54	5.36	5.35
Dexamethasone sodium phosphate	5.43	5.43	5.34	5.33
Dopamine hydrochloride	5.30	5.29	5.30	5.34
Dobutamine hydrochloride	5.30	5.30	5.29	5.34
Furosemide	5.36	5.37	5.30	5.30
Carbazochrome sodium sulfonate hydrate	5.37	5.36	5.34	5.33
Tranexamic acid	5.43	5.43	5.35	5.33
Haloperidol	5.34	5.36	5.31	5.32
Metoclopramide hydrochloride	5.38	5.37	5.33	5.33
Octreotide acetate	5.38	5.39	5.33	5.31
Lansoprazole	5.42	5.44		
Granisetron hydrochloride	5.37	5.37		
Ramosetron hydrochloride	5.37	5.37		
Omeprazole sodium hydrate	5.42	5.44		
Heparin sodium			5.34	5.33
Calcium chloride hydrate			5.31	5.29
Magnesium sulfate hydrate			5.31	5.30

Model A: the fat emulsion and the therapeutic agent administered through the side tube of the central venous line; model B: the fat emulsion administered through the lateral tube of the TPN infusion product mixed with a therapeutic agent; gray area: not done in this experiment; 0 h: the pH value immediately after preparation; 24 h: the pH value 24 h after preparation.
